# Metformin enhances nitric oxide production and diminishes Rho kinase activity in rats with hyperlipidemia

**DOI:** 10.1186/1476-511X-13-115

**Published:** 2014-07-15

**Authors:** Yan Liu, Congwu Huang, Chuan Ceng, Haiyong Zhan, Dongdan Zheng, Weixing Han

**Affiliations:** 1 The First Affiliated Hospital of Anhui Medical University, Hefei, Anhui Province, China; 2 The Department of Internal Medicine, the Second Affiliated Hospital of Shantou University, Shantou, Guangdong Province, China; 3 The Department of Cardiology, The First Affiliated Hospital of Sun Yat-sen University, Guangzhou, Guangdong Province, China; 4 The Third people's hospital of Huizhou, Huizhou, Guangdong Province, China

**Keywords:** Hyperlipidemia, Nitric oxide, Rho kinase

## Abstract

**Background:**

Rho kinase over-activation is associated with nitric oxide (NO) reduction and atherosclerosis. Metformin is favorable for endothelial function improvement and cardiovascular outcomes. Whether cardio-protective effect of metformin is associated with Rho kinase activity is unknown.

**Methods:**

Hyperlipidemia model of rats were established accordingly. Thereafter, medical interventions in terms of atorvastatin, metformin or combined therapy were administered for 4 weeks. Laboratory parameters were compared among each groups at initial, 6 weeks of high-fat and high-cholesterol diet administration, and 4 weeks of medical intervention. Lineal regression analyses were performed.

**Results:**

No significant difference of laboratory parameters was observed initially. Six weeks of high-fat and high-cholesterol diet administration, serum levels of cholesterol, C-reactive protein (CRP) level, and Rho kinase activity were significantly increased while NO production was concomitantly reduced in comparison to the sham group. After 4 weeks of medical intervention, CRP level and Rho kinase activity were profoundly diminished while NO production was significantly enhanced in the atorvastatin and metformin groups, and these benefits were further enhanced with combined therapy. Lineal regression analyses showed that Rho kinase activity was negatively correlated with NO production but positively correlated with CRP level.

**Conclusion:**

In rats with hyperlipidemia, metformin and atorvastatin therapy is favorable for NO production and CRP reduction, which might be associated with Rho kinase activity decrease.

## Introduction

Atherosclerosis and its associated cardiovascular diseases (CVD) are still the leading cause of morbidity and mortality worldwide [1]. Accordingly, hyperlipidemia, hypertension, diabetes mellitus, smoking and so on are the major causes of atherosclerosis and CVD. Previously, many basic and clinical studies have demonstrated that dyslipidemia improved by lipid lowering drugs such as statins is associated with decreased risk of cardiovascular events such as ischemic stroke and acute myocardial infarction [2-5]. Notably, in subjects with normal lipid level, statins therapy also have robust protective effects on cardiovascular system which currently known as statins’ pleiotropic effects [6]. Statins’ pleiotropy includes anti-inflammation, anti-oxidation, vascular-protection as well as promotion of endothelial progenitor cells migration. Accordingly, these pleiotropic effects of statins are associated with inhibition of isoprenylation of small GTP-binding proteins such as Ras and Rho [6-8]. Small GTP-binding proteins are important intermediates which generate in the process of cholesterol biosynthesis, and have complex physiological and pathological effects such as regulating cell’s mobility, migration, proliferation, survival and apoptosis [8,9]. Importantly, many studies have shown that increased activity of small GTP-binding proteins was associated with increased risk of atherosclerosis and CVD [10,11], and statins’ potent cardio-protective efficacy was largely dependent on inhibiting these small GTP-binding proteins isoprenylation rather than lipid-lowering [12,13].

Metformin is a classic medication for diabetes treatment. Previously, some studies have shown that metformin therapy was favorable for cardiovascular outcomes, and the mechanisms might be partially associated with its effects on improving insulin resistance, reducing serum level of C-reactive protein (CRP), promoting endothelial nitric oxidase synthesis (eNOS) expression and nitric oxide (NO) production and regulating glucose metabolism [14-16]. As is well known that atherosclerosis is a chronic inflammation status and characterized by endothelial dysfunction, increased systemic inflammation, disorder of cholesterol and glucose metabolism [17]. Since hyperlipidemia is detrimental to endothelium and is capable of eliciting systemic inflammation as reported by previous published articles [18,19], we therefore considered that in light of its aforementioned cardio-protective effects, metformin might be possible to further enhancing statin effects on ameliorating inflammation and improving endothelial function. Moreover, with regard to the adverse effects of small GTP-binding proteins over-activation under pathological condition, we further investigated whether metformin could modulate small GTP-binding proteins activity in the setting of hyperlipidemia.

## Methods

### Hyperlipidemia model production and medical intervention

Our current study was approved by Ethic Committee of Anhui Medical University. Totally 50 male Sprague–Dawley (SD) rats weighing 200-220 g were used in our current study (obtained from Experimental Animal Center of the First Affiliated Hospital of Anhui Medical University, Hefei, Anhui Province, China). Forty rats were used to produce hyperlipidemia model and 10 rats were served as sham group. The protocol for hyperlipidemia model establishment was in accordance to our previous published study (cholesterol 4.0%, cholic acid 0.4%, propylthiouracilum 0.3% and lard 10.0%) [20], and the high-fat and high-cholesterol diet was given for 6 weeks. After hyperlipidemia model was successfully established as assessed by serum levels of lipid profile, medical intervention was performed for 4 weeks and the protocol was as follow: 32 hyperlipidemia rats were randomly and evenly divided into 4 groups named control group (orally given 3 ml normal saline), statins group (orally given atorvastatin 10 mg/kg body weight/day, reconstituted in 3 ml normal saline), metformin group (orally given metformin 50 mg/kg body weight/day, reconstituted in 3 ml normal saline), and combined group orally given atorvastatin and metformin with the same dosages as described above.

### Laboratory examination

At the initial of this study, at 6 weeks of hyperlipidemia establishment and 4 weeks of medical intervention, fasting blood sample in each group was drawn for laboratory parameter examination. Lipid profiles including triglyceride (TG), total cholesterol (TC), low density lipoprotein cholesterol (LDL-C), and high density lipoprotein cholesterol (HDL-C), and liver enzymes such as alanine aminotransferase (ALT) and aspartate aminotransferase (AST), serum fasting blood glucose (FBG), CRP and creatinine kinase (CK) levels were evaluated by Automatic Biochemistry Analyzer (Hitachi 7150, Tokyo, Japan). Serum Rho kinase activity was detected by enzyme-linking immune-absorbent assay (ELISA, Yuping BioMedical Company, Shanghai, China), and the range varies from 20 U/L to 800 U/L. Procedure was performed strictly in accordance to the manufacture’s manual. Serum level of nitric oxide (NO) was evaluated by nitrite reductase method using Total Nitric Oxide Kit (Beyotime, Haimen, China, S0023). Three independent experiments were performed in duplicate.

### Statistical analyses

All continuous variables were expressed as mean ± SD, and analyses were performed with SPSS software, version 18.0 (SPSS Science, Chicago, IL, USA). Statistical significance among groups was evaluated with One Way ANOVA (post hoc LSD-t). Where linear regression was used, the Pearson or Spearman correlation coefficient was reported, and a value of *P* < 0.05 was considered statistically significant.

## Results

### Effects of metformin and atorvastatin on laboratory parameters

As presented in Table 1, laboratory parameters at initial among each group were comparable. Six weeks of high-fat and high-cholesterol diet administration, hyperlipidemia model was successfully established as evidenced by the serum levels of lipid profile were significantly increased when compared to the sham group. Additionally, serum level of CRP was also profoundly increased in the hyperlipidemia groups. With 4 weeks of medical intervention, serum levels of TG, TC and LDL-C were reduced in the atorvastatin and combined groups, whereas metformin therapy had no effects on lipid profiles improvement. Nevertheless, with 4 weeks of metformin administration, serum level of CRP was significantly decreased when compared to the control group. Although serum level of CRP was a little bit lower in the atorvastatin group than that of the metformin group, no significant difference was observed (5.18 ± 0.98 mg/L versus 5.27 ± 0.78 mg/L, P = 0.137). Notably, when metformin was added into atorvastatin therapy, serum level of CRP was further diminished (4.53 ± 0.73 mg/L, P < 0.05 versus atorvastatin and metformin groups). Moreover, with 6 weeks of high-fat and high-cholesterol diet administration, serum levels of FBG in hyperlipidemia groups were slightly increased in comparison to the sham group, and with 4 weeks of medical intervention, a modest improvement of FBG was observed in the metformin and combined groups, whereas no improvement was found in the atorvastatin and control groups. There were no significant differences of ALT, AST, and CK among these groups.

**Table 1:** Effects of metformin and atorvastatin on laboratory parameters

**Variables**	**Sham**	**Control**	**Atorvastatin**	**Metformin**	**Combined**
**Initially**					
**TG (mmol/L)**	0.97 ± 0.10	0.96 ± 0.12	0.95 ± 0.12	0.96 ± 0.11	0.94 ± 0.12
**TC (mmol/L)**	3.12 ± 0.28	3.15 ± 0.30	3.18 ± 0.32	3.16 ± 0.32	3.19 ± 0.29
**LDL-C (mmol/L)**	1.97 ± 0.20	1.98 ± 0.20	1.99 ± 0.22	1.96 ± 0.18	2.00 ± 0.18
**HDL-C (mmol/L)**	1.05 ± 0.05	1.07 ± 0.04	1.06 ± 0.04	1.06 ± 0.05	1.06 ± 0.05
**CRP (mg/L)**	1.34 ± 0.18	1.37 ± 0.15	1.36 ± 0.15	1.35 ± 0.17	1.37 ± 0.16
**ALT (U/L)**	26.2 ± 3.2	26.7 ± 3.1	27.2 ± 2.9	27.1 ± 2.4	26.8 ± 2.2
**AST (U/L)**	27.7 ± 2.6	27.9 ± 2.1	28.0 ± 2.5	28.3 ± 3.0	27.8 ± 2.0
**CK (U/L)**	13.3 ± 1.4	13.7 ± 1.2	14.2 ± 1.0	14.0 ± 1.2	13.5 ± 1.1
**FBG (mmol/L)**	4.64 ± 0.37	4.57 ± 0.32	4.68 ± 0.30	4.66 ± 0.33	4.74 ± 0.25
**6 weeks later**					
**TG (mmol/L)**	0.99 ± 0.10*	2.18 ± 0.31	2.17 ± 0.30	2.18 ± 0.22	2.18 ± 0.21
**TC (mmol/L)**	3.11 ± 0.29*	5.87 ± 0.57	5.90 ± 0.60	5.93 ± 0.62	5.91 ± 0.59
**LDL-C (mmol/L)**	1.95 ± 0.18*	3.83 ± 0.53	3.84 ± 0.51	3.82 ± 0.50	3.79 ± 0.50
**HDL-C (mmol/L)**	1.08 ± 0.05	1.09 ± 0.05	1.08 ± 0.04	1.08 ± 0.04	1.09 ± 0.05
**CRP (mg/L)**	1.35 ± 0.12*	7.87 ± 1.02	7.90 ± 1.06	7.88 ± 1.05	7.92 ± 1.02
**ALT (U/L)**	28.4 ± 3.0	29.7 ± 3.1	29.7 ± 2.2	28.8 ± 1.0	28.8 ± 1.6
**AST (U/L)**	28.0 ± 3.1	28.5 ± 2.2	28.1 ± 2.1	28.4 ± 2.2	28.4 ± 1.6
**CK (U/L)**	13.9 ± 1.2	13.2 ± 1.0	14.0 ± 1.1	13.7 ± 1.2	13.3 ± 1.5
**FBG (mmol/L)**	4.79 ± 0.42	5.83 ± 0.38	5.86 ± 0.35	5.92 ± 0.35	5.81 ± 0.28
**4 weeks of intervention**					
**TG (mmol/L)**	0.97 ± 0.11*	2.19 ± 0.21	1.93 ± 0.10	2.17 ± 0.18	1.93 ± 0.12
**TC (mmol/L)**	3.02 ± 0.22*	5.74 ± 0.36#	4.60 ± 0.38	5.79 ± 0.37	4.52 ± 0.37
**LDL-C (mmol/L)**	1.91 ± 0.15*	3.72 ± 0.26#	2.91 ± 0.28	3.73 ± 0.33	2.86 ± 0.16
**HDL-C (mmol/L)**	1.07 ± 0.04	1.08 ± 0.04	1.09 ± 0.03	1.09 ± 0.03	1.10 ± 0.04
**CRP (mg/L)**	1.36 ± 0.10*	7.22 ± 1.06#	5.18 ± 0.98	5.27 ± 0.78	4.53 ± 0.73&
**ALT (U/L)**	27.6 ± 2.1	27.9 ± 1.1	28.5 ± 2.2	28.2 ± 1.1	28.0 ± 2.1
**AST (U/L)**	27.9 ± 2.2	28.2 ± 2.0	28.6 ± 2.1	28.3 ± 1.8	27.9 ± 1.2
**CK (U/L)**	14.6 ± 1.1	13.8 ± 1.2	14.3 ± 1.2	14.2 ± 1.1	13.9 ± 1.2
**FBG (mmol/L)**	4.77 ± 0.46	5.71 ± 0.45	5.72 ± 0.43	4.98 ± 0.39	5.05 ± 0.45

Denote: *P < 0.05 sham group versus other group, #P < 0.05 control group versus atorvastatin and combined groups, &P < 0.05 combined group versus atorvastatin and metformin groups.

### Effects of metformin and atorvastatin on NO production and Rho kinase activity

**Figure 1:**
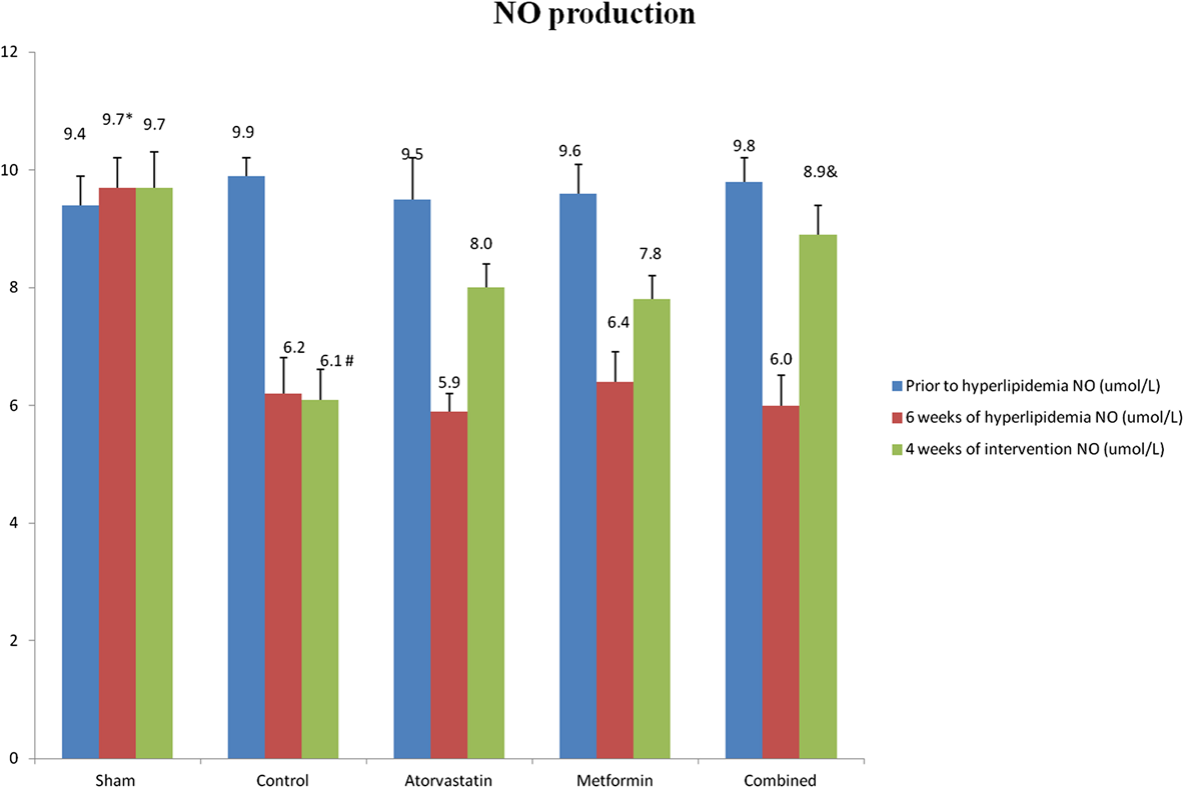
Denote: *P < 0.05 versus other groups at 6 weeks of hyperlipidemia model production, #P < 0.05 versus atorvastatin, metformin and combined groups, &P < 0.05 versus atorvastatin and metformin groups.

**Figure 2:**
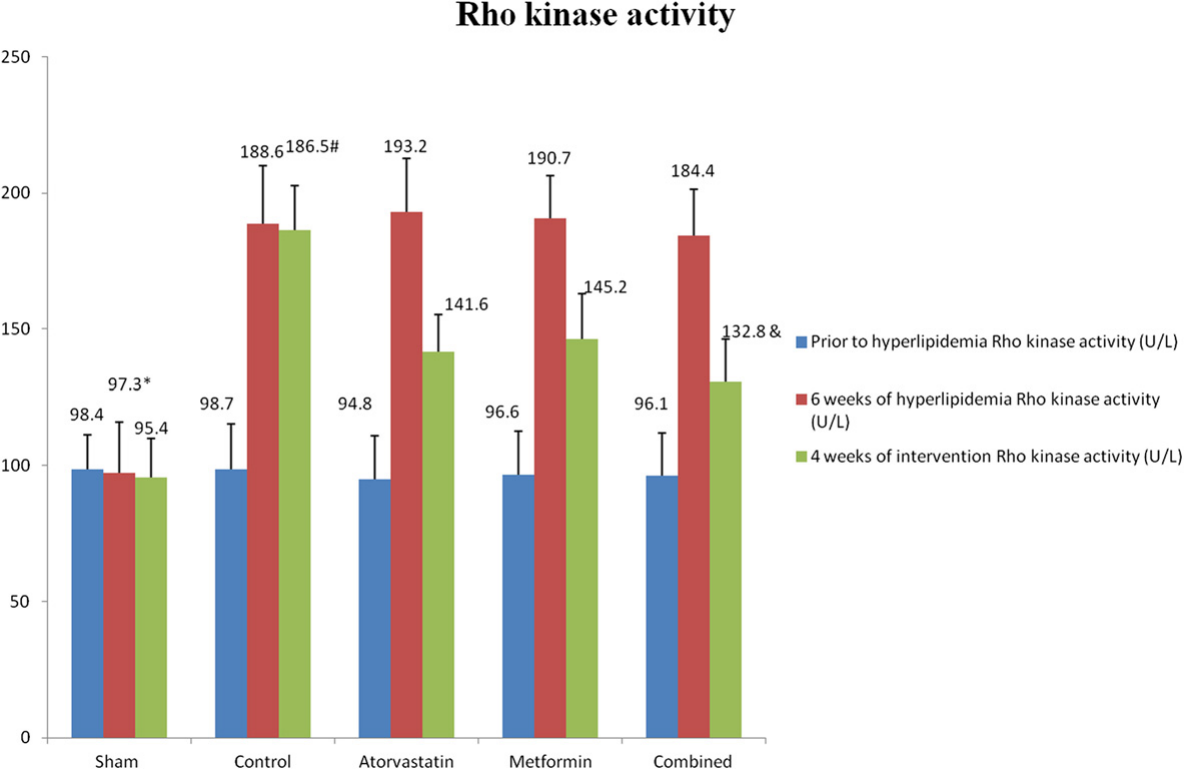
Denote: *P < 0.05 versus other groups at 6 weeks of hyperlipidemia model production, #P < 0.05 versus atorvastatin, metformin and combined groups, &P < 0.05 versus atorvastatin and metformin groups.

As shown in Figures 1 and 2, 6 weeks of high-fat and high-cholesterol diet administration, NO production was profoundly declined in the hyperlipidemia groups in comparison to the sham group (P < 0.05). In contrast, Rho kinase activity was elevated in all hyperlipidemia groups (P < 0.05 versus sham group). With 4 weeks of metformin and atorvastatin therapy, NO production was increased in accompany with Rho kinase activity decrease in the atorvastatin and metformin groups, and these efficacies were further enhanced with combined therapy (P < 0.05 versus atorvastatin and metformin groups). Notably, with 4 weeks of medical intervention, the magnitude of Rho kinase activity decrease appeared to be more significant in the combined groups (132.8 ± 15.5 U/L) than that of the metformin (145.2 ± 16.7 U/L) and atorvastatin group (141.6 ± 13.7 U/L) but without statistical significance.

### Linear regression analyses

**Figure 3:**
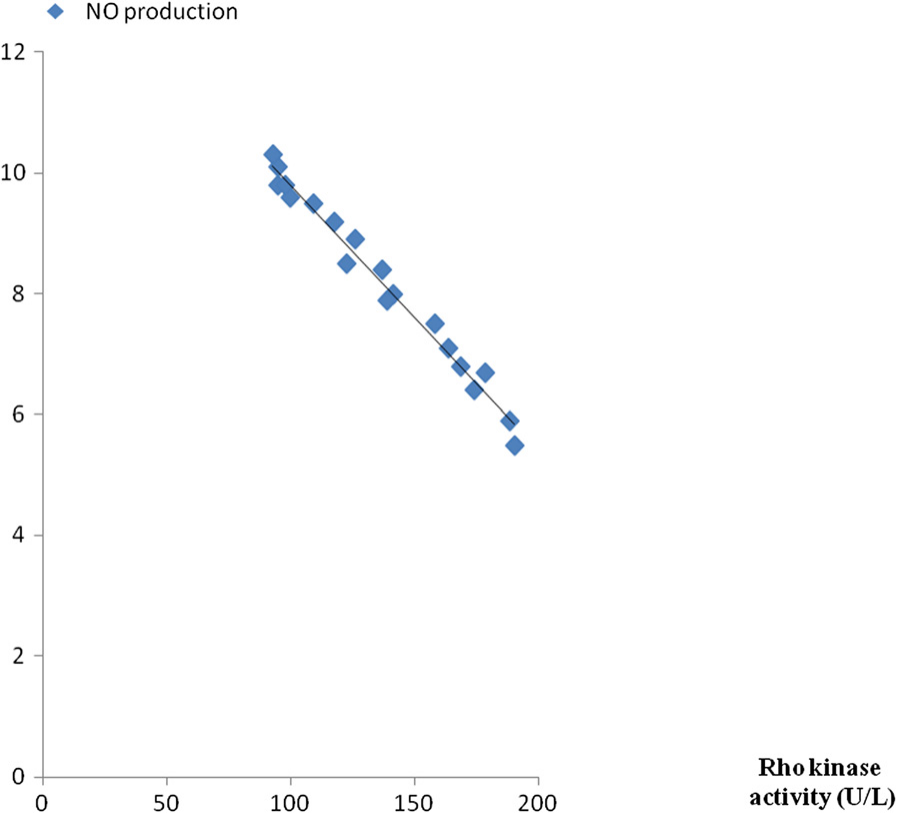
Correlation analyses: r = −0.608, P < 0.001.

**Figure 4:**
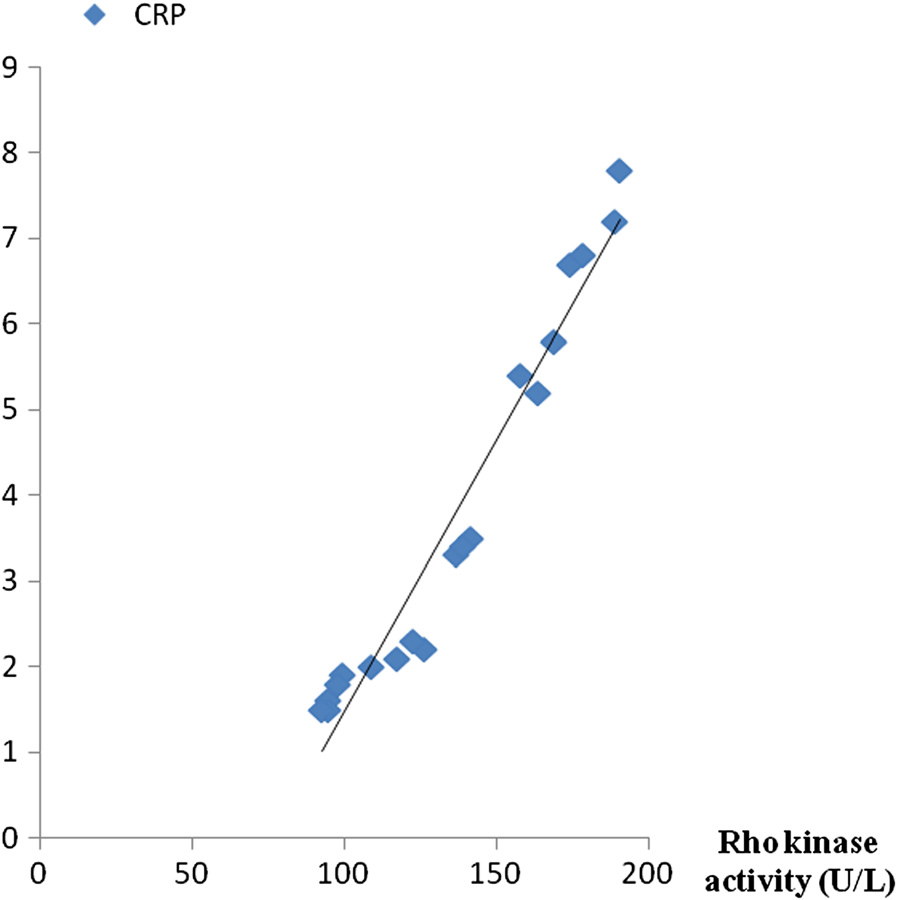
Correlation analyses: r = 0.636, P < 0.001.

In order to investigate the relationship between laboratory parameters, linear regression analyses were performed. As revealed in Figures 3 and 4, Rho kinase activity was inversely correlated with NO production (r = − 0.608, P < 0.001) while was positively correlated with CRP level (r = 0.636, P < 0.001). Additionally, linear regression analyses showed no significant correlation between Rho kinase activity and other variables such as LDL-C and FBG.

## Discussion

Results from our current study indicate that in rats with hyperlipidemia, either metformin or atorvastatin treatment is favorable for reducing CRP level, increasing NO production and decreasing Rho kinase activity. These cardio-protective effects are further enhanced by combined therapy with metformin and atorvastatin. Notably, metformin has no effects on lipid profile change, indicating that these benefits derived from metformin therapy may be associated with other mechanisms such as declining Rho kinase activity in the setting of hyperlipidemia since linear regression analyses show that Rho kinase activity is positively correlated with CRP level while negatively correlated with NO production in the metformin group.

Accordingly, the benefits of statins therapy can be categorized into two respects in terms of lipid-lowering efficacy and pleiotropic effects. Notably, statins’ pleiotropy is largely associated with its potent effect on inhibiting small GTP-binding proteins isoprenylation. With isoprenylation, small GTP-binding proteins such as Rho can play multiple and complex roles on endothelium, leukocyte and fibroblast. Under pathological setting such as hyperlipidemia and diabetes mellitus, Rho isoprenylation and its effector Rho kinase (ROCK) over-activation contribute detrimental effects on vascular system such as promoting inflammatory cells adhesion and infiltration, impairing endothelial function, increasing reactive oxidative species generation, destabilizing eNOS mRNA and decreasing NO production. Findings from our current study further corroborated previous reports that in the setting of hyperlipidemia, statins treatment not only could improve dyslipidemia, but also concomitantly decreased Rho kinase activity which consequently reduced CRP level and enhanced NO production [8,21].

Interestingly and importantly, our current study also preliminarily suggested that in the setting of hyperlipidemia, 50 mg/Kg body weight per day of metformin administration was beneficial for NO production increment and CRP level reduction which was consistent to previous studies [14,22,23]. Nevertheless, the mechanisms associated with these benefits are still incompletely clear. In light of our current study, we speculated that the benefits derived from metformin therapy of rats with hyperlipidemia might be partially associated with its effects on inhibiting Rho kinase activity. Nevertheless, since metformin had no effects on cholesterol biosynthesis as revealed in our study, inhibiting Rho kinase activity of metformin might not be related to its inhibition of isoprenylation of small GTP-binding proteins. Rather, on the basis of previous studies [14,24,25], we considered that some alternative mechanisms might be associated with diminishment of Rho kinase activity with metformin therapy. First of all, basic researches suggest that cardio-protective effects of metformin are partially mediated by AMP-activated protein kinase (AMPK) activation which is responsible for eNOS up-regulation and NO production increase [14,26]. Accordingly [27,28], Rho kinas activated is partially dependent on reactive oxidative species, and up-regulation of eNOS expression and NO production ameliorate oxidative stress therefore inhibiting Rho kinase activation. Secondly, since high blood glucose is a potential stimulus for Rho kinase activation, and Rho isoprenylation and Rho kinase over-activation contribute to insulin resistance which reciprocally induces high blood glucose [25,29], therefore we considered that it was possible that metformin declined Rho kinase activity by means of improving glucose metabolism. In our current study, we observed that rats with high-fat and high-cholesterol diet administration for 6 weeks, fasting blood glucose was somewhat increased in the hyperlipidemia groups than that of the sham group, indicating that hyperlipidemia per se might potentially compromise glucose metabolism. Nevertheless, with 4 weeks of metformin therapy, fasting blood glucose levels in the metformin and combined groups were reduced in comparison to the control and atorvastatin groups, suggesting that metformin therapy was favorable for improving glucose metabolism despite in non-diabetes condition. Although all the hyperlipidemia groups were not qualified to the criteria of diabetes mellitus, we considered that increased fasting blood glucose might be associated with Rho kinase activity enhancement, and in the metformin and combined groups, Rho kinase activity was a little bit lower than that of the atorvastatin group which might directly suggest that fasting blood glucose improvement with metformin therapy was favorable for declining Rho kinase activity.

Importantly, favorable effects in terms of CRP reduction, NO production, and Rho kinase activity decrease were more robust in the combined therapy groups, suggesting that atorvastatin and metformin might have synergistic protective effects. In light of previous reports and our current findings [23,30-32], we considered that these benefits might derive from anti-inflammation and lipid-modification of atorvastatin as well as anti-inflammation and glucose-metabolism improvement of metformin which concomitantly leaded to Rho kinase activity diminishment.

No liver enzymes and creatinine kinase elevation were observed with four weeks of metformin and atorvastatin therapy, suggesting the high safety profile of current combined therapeutic strategy.

## Conclusion

Our current study indicates that in rats with hyperlipidemia, metformin and atorvastatin therapy is favorable for NO production and CRP reduction, which might be associated with Rho kinase activity decrease.

## Competing interests

The authors declare that they have no competing interests.

## Authors’ contributions

CC, HZ and YL performed this study, DZ designed this study and performed statistic analyses, CH wrote this article, YL and WH helped to revise the final version of paper. All authors read and approved the final manuscript.

## Authors’ information

Yan Liu and Congwu Huang: co-first authors.
